# Hope for the Dental Profession. The Cummings' Patent

**Published:** 1878-07

**Authors:** 


### EDITORIAL, ETC.

[The following circular has been recently issued. The profession is familiar with the hard fight made against this monopoly in the past, and familiar also with the results. The new evidence here presented is certainly strong corroboration of the conviction which all fair-minded men have held from the start, that the Goodyear Dental Vulcanite Company had no just right to the royalty they collected. The importance of the matter to the profession is seen in the strenuous efforts of those who are working up this new case, and the previous failures made may in this case show the way to success, and if it can be done, it is the duty of the Dentists to shake off this oppression. The fact cited in the circular respecting the use the Rubber Company intend making of the Hæring patent should stimulate endeavor to break their case. We trust that entire success will attend this latest effort.—Ed. Amer. Jour. Dental Science.]

*Hope for the Dental Profession.*—At last, after so many cruel disappointments, there is a strong hope grounded in facts that the Dental Profession will be relieved from the odium of a long subjection to the unjust monopoly of an illegal patent.

For twelve years its members have been forced to pay to the Cummings patent, in the hands of the Goodyear Dental Vulcanite Company, a tribute amounting to millions of dollars.

They have during this time made many attempts to throw off this galling yoke, but their efforts were fruitless, because in every case there was either treachery and collusion on the part of those whom they trusted, or a failure to place the strong points of the defence in a proper manner before the Courts, and to sustain these strong points by competent proof that was in existence and accessible.

This announcement of the cause of former defeats was made a year and a half ago in a circular that was sent to prominent Dentists in all parts of the country. The position then taken is now fully confirmed by the testimony for the defence, already taken and on record, in the cause of the Goodyear Dental Vulcanite Company *vs.* O. H. Brightwell. This is one of several suits instituted in July, 1876, against Dentists in Baltimore and Washington, for infringement of the Cummings patent. It is now being rapidly pushed to a conclusion, and has progressed so far that the direct testimony for the defence is completed and on record. This defence, with the testimony supporting it. is essentially different in form and matter from that of any former hearin.

Our proof as recorded is:

1st. That when John A. Cummings made his application for a patent in 1855, and even when he filed his caveat in 1852, there was a patent on record in the Patent Office which covered all the ground that he claimed or could claim, or in nearly the officical language of that office, "deprived his invention of all claim to novelty."

2nd. That prior to 1852, dental plates, both experimental and practical, were made of hard rubber, thus establishing priority of invention over him by antedating him, even if he did invent what he claimed.

3rd. That he did not invent what he claimed, for we have evidence that he knew nothing practically about making dental plates of hard rubber until 1861 or 1862, when he paid an agent of the American Hard Rubber Company, who testifies to that fact, to instruct him in the method and process of making such plates; also, that the man from whom he obtained the model filed by him in 1855, could not at that time make such plates.

4th. That after the rejection of his application in 1856, this patent became an abandoned one by the operation of the laws and rules governing the Patent Office, as well as by the practice thereof, and more especially and fully by his personal knowledge of the general use of hard rubber for dental plates between the years 1856 and 1864, without protest or objection on his part to such use as an infringement of his rights, and further by his express declaration that it was a humbug, and that he would have nothing more to do with it.

The evidence on these points is direct and positive, and taken on a whole, forms as we believe, an impregnable structure of defence. It has been obtained and placed on record only by great care and unceasing watchfulness on the part of the attorney and the representative of the Dental Profession who have been conducting the defence. The greater part of the labor has been performed, but much that is important remains to be done. The Attorney must attend the taking of testimony by the complainants in rebuttal, and cross-examine the witnesses; the case must be made up for the hearing, and the record printed. This of course cannot be done without further expense, and we ask every member of the Profession who sees this circular, to join with those who have so liberally subscribed, and aid in the vindication of the Profession from this great and shameful wrong, a wrong which is not limited to the existence of the Cummings patent, but will, if we are not now successful, continue for five years after. For the Goodyear Dental Vulcanite Company claim another patent, the "Hæring," which is almost identical with the Cummings, and which—issued in 1869—does not expire till 1886. We have received intimation that the Goodyear Dental Vulcanite Company intend to enforce this patent as they have the Cummings; they have indeed licensed under the Cummings *and Hœring* patents.

The former decisions of the United States District and Supreme Courts were based upon *the evidence as presented in the cases then*

*before them*, and cannot be conclusive upon the present one, in which a different state of facts is presented and proven in support of a different and stronger line of defence.

This great case is thus briefly stated, and the views set forth in the statement are endorsed, with but few exceptions, by all who have become acquainted with the character and directness of the evidence, including many of the most intelligent Dentists in New York, Boston, Baltimore and Washington, because they are satisfied from the results thus far attained, that the work in this case has been and will be to the end *honest* and *earnest.*

Then let every Dentist in the land do his part to aid in this last effort which gives such fair promise of being successful, to free the Dental Profession from the merciless clutches of Josiah Bacon & Co.

Contributions will be received and acknowledged by either of the undersigned, and the last named, Dr. Hunt, will cheerfully give to those desiring it, all information he can on the subject.

Dr. W. H. Dwinelle, Chairman N. Y. Committee, 27 W. 34th Street, New York.

Dr. W. H. Allen, Treasurer N. Y. Committee, 18 W. 11th Street, New York.

Dr. R. B. Winder, Baltimore Committee, 140 Park Avenue, Baltimore.

Dr. L. D. Shepard, 100 Boylston Street, Boston.

Dr. R. Finley Hunt, Manager for Defendants, 1806 H. St., Washington, D. C.

